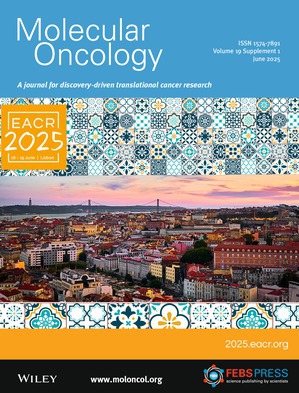# Abstracts

**DOI:** 10.1002/1878-0261.70070

**Published:** 2025-06-11

## Abstract

Abstracts submitted to the ‘EACR 2025 Congress: Innovative Cancer Science’, from 16–19 June 2025 and accepted by the Congress Organising Committee are published in this Supplement of *Molecular Oncology*, an affiliated journal of the European Association for Cancer Research (EACR).